# Minimally invasive esophagectomy in the semi-prone position for corrosive esophagitis: a case report

**DOI:** 10.1093/jscr/rjac218

**Published:** 2022-07-30

**Authors:** Tamotsu Obitsu, Hirokazu Kiyozaki, Masaaki Saito, Shota Fukai, Iku Abe, Kosuke Ichida, Yuta Muto, Toshiki Rikiyama

**Affiliations:** Department of Surgery, Saitama Medical Center, Jichi Medical University, Saitama, Japan; Department of Surgery, Saitama Medical Center, Jichi Medical University, Saitama, Japan; Department of Surgery, Saitama Medical Center, Jichi Medical University, Saitama, Japan; Department of Surgery, Saitama Medical Center, Jichi Medical University, Saitama, Japan; Department of Surgery, Saitama Medical Center, Jichi Medical University, Saitama, Japan; Department of Surgery, Saitama Medical Center, Jichi Medical University, Saitama, Japan; Department of Surgery, Saitama Medical Center, Jichi Medical University, Saitama, Japan; Department of Surgery, Saitama Medical Center, Jichi Medical University, Saitama, Japan

## Abstract

Treatment strategies for corrosive esophagitis include conservative treatment, such as balloon dilatation at the stenosis site, and surgical treatment. Esophagectomy for corrosive esophagitis is usually performed through the transthoracic or transhiatal approaches. Herein, we report a case of corrosive esophagitis treated with thoracoscopic esophagectomy with the patient in the semi-prone position. The patient was a 48-year-old woman who developed corrosive esophagitis due to accidental ingestion of an alkaline agent. Surgical intervention was required for esophageal stenosis. Therefore, thoracoscopic esophagectomy was performed with the patient in the semi-prone position with bilateral pulmonary ventilation. In our hospital, good operative outcomes have been obtained using thoracoscopic esophagectomy for esophageal cancer with the patient in the semi-prone position with bilateral pulmonary ventilation. This technique is also considered effective for the treatment of corrosive esophagitis.

## INTRODUCTION

Suicide attempts or accidental ingestion of corrosive substances, such as a strong acid or strong alkali, can cause liquefactive necrosis in the deep part of the upper gastrointestinal tract tissue within a short period of time and can also cause perforation. Delayed onset gastrointestinal stenosis may also occur due to the formation of fibrotic scarring, even during the healing process [1].

In cases of gastrointestinal stenosis caused by a corrosive substance, conservative therapy, such as endoscopic balloon dilatation, is indicated in the absence of severe stenosis. However, in cases of extensive stenosis, improvement of symptoms cannot be expected using conservative treatment, and surgery becomes necessary [2].

There have been many recent reports of minimally invasive esophagectomy (MIE), a thoracoscopic approach, for corrosive esophagitis [3]. However, there are no past reports of the procedure being performed in patients in the semi-prone position. Herein, we describe a new approach for the treatment of corrosive esophagitis using thoracoscopic esophagectomy with the patient in the semi-prone position.

**Figure 1:**
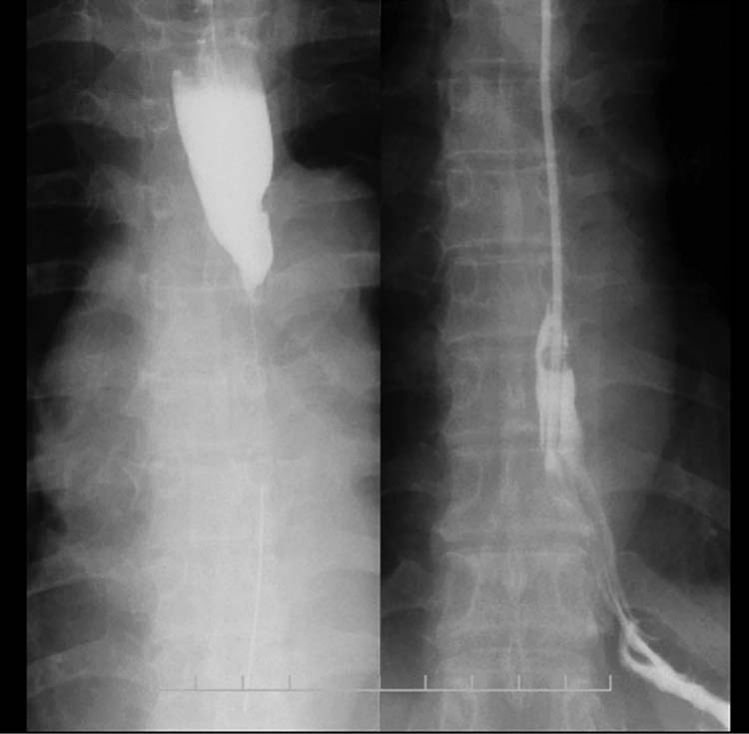
Esophagram findings; the esophagram shows stricture in the middle thoracic esophagus; a nasogastric tube could pass through the stenosis, and tube feeding was started.

## CASE REPORT

A 48-year-old woman accidentally ingested an alkaline detergent and was transported to the emergency medical care center of a local hospital. Conservative treatment using a proton pump inhibitor and a mucosal protectant was initiated. Although this treatment was lifesaving, an esophageal stricture developed, and the patient developed dysphagia and gradually lost weight. Two months later, she was referred to our hospital for balloon dilatation. The esophageal stenosis was severe and >10 cm in length (Fig. 1); therefore, balloon dilatation was impossible. A nasogastric tube was inserted to improve the patient’s nutritional status. After 3 months, her nutritional status improved, and surgical treatment was deemed necessary.

**Figure 2:**
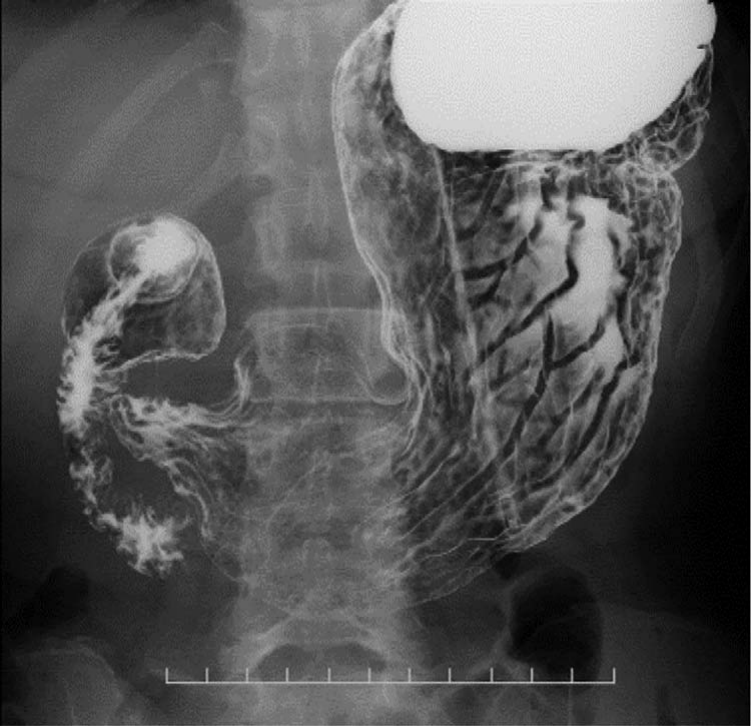
CT images showing a horizontal section of the esophagus; CT image shows circumferential wall thickening with infiltrative changes in the middle part of the thoracic esophagus.

**Figure 3:**
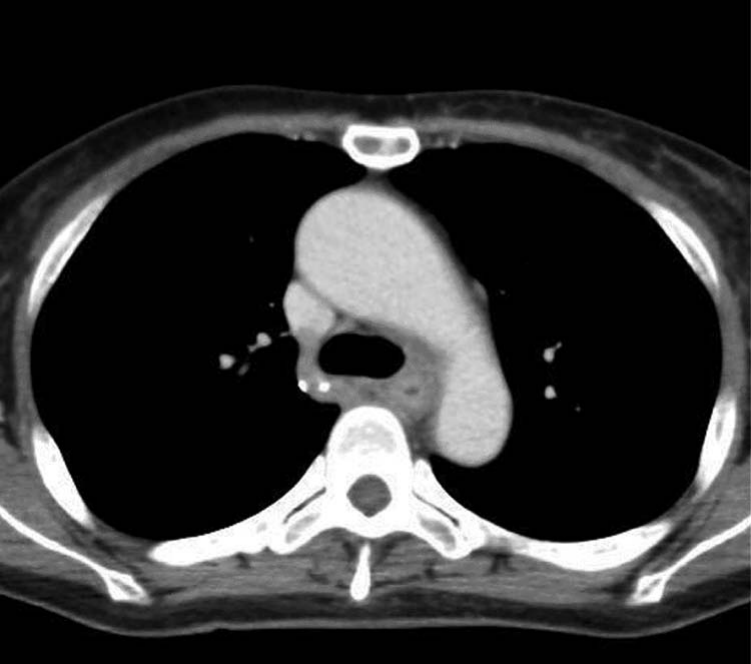
Coronal plane of the CT findings; CT demonstrates dilatation of the upper thoracic esophagus.

**Figure 4:**
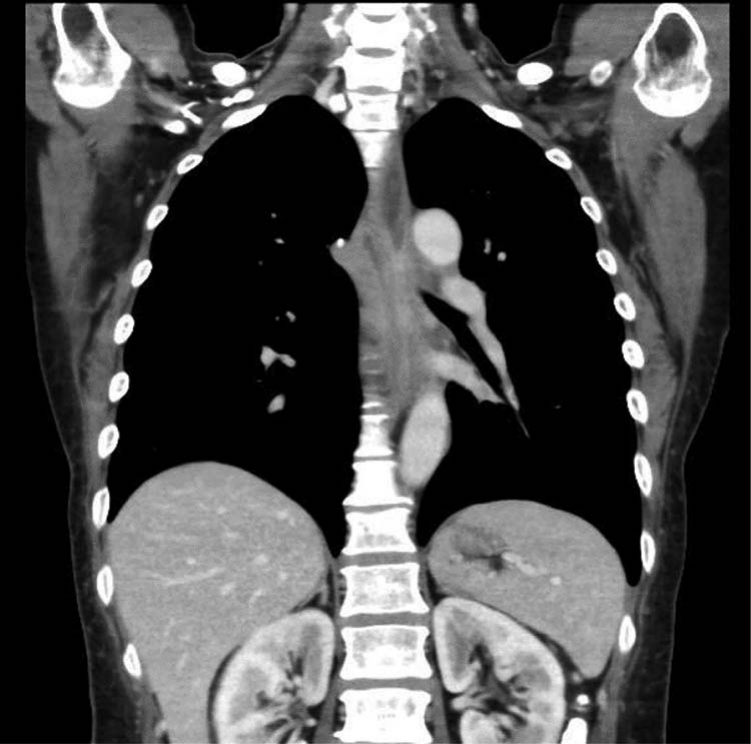
Upper gastrointestinal series showing the stomach; there were no specific findings in the stomach.

**Figure 5:**
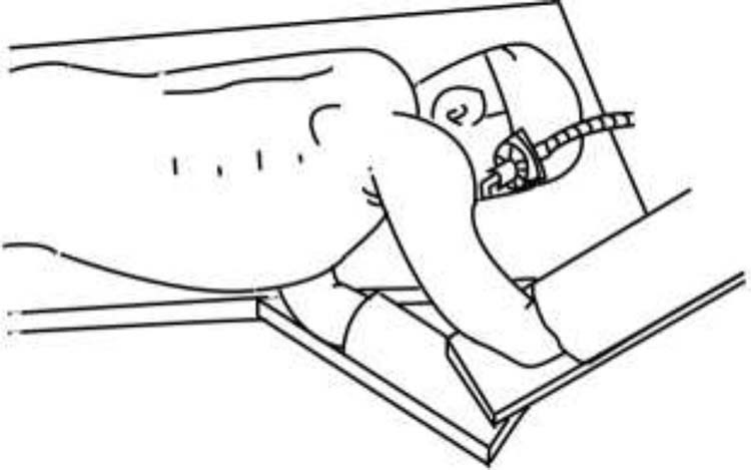
Semi-prone position; in an emergency, this position allows for a rapid transition to the left lateral decubitus position using bed rotation.

Preoperative computed tomography (CT) and barium meal studies were performed. The CT findings showed circumferential wall thickening with infiltrative change at the middle of the intrathoracic esophagus, with luminal dilation of the oral side of the stenotic esophagus (Figs 2, 3). The barium meal study showed no abnormal findings in the stomach (Fig. 4). Thoracoscopic esophagectomy was performed with the patient in the semi-prone position, followed by esophageal reconstruction using a gastric tube with the patient in the supine position. The operation was started with bilateral lung ventilation using a single-lumen endotracheal tube. A four-port thoracoscopic technique was used as follows: an observation port with a 10-mm scope was placed at the ninth intercostal space at the line of the inferior scapular angle, and two 5-mm ports and one 12-mm port for the intrathoracic procedures were placed at the third, fifth and seventh intercostal spaces, respectively, at the posterior axillary line (Fig. 5). Pneumothorax was created using 10 mmHg of CO_2_ to deflate the lung to achieve a better operative field. The pleura and connective tissue around the thoracic esophagus showed severe inflammatory and fibrotic changes. In particular, the middle thoracic esophagus was strongly adherent to the arch of the azygos vein and the tracheal bifurcation.

A subtotal gastric tube was created through a small upper abdominal incision and pulled up to the neck through the retrosternal route. Cervical esophagogastric anastomosis was performed using the modified Collard method [4] using a linear stapler. We then performed tube jejunostomy for enteral nutrition and tube gastrostomy for decompression of the gastric tube.

Minor anastomotic leakage was observed on postoperative Day 7, but the patient recovered with conservative treatment. The patient was discharged on postoperative Day 16. She received outpatient follow-up at our hospital for 2.5 years.

## DISCUSSION

Many cases of corrosive esophagitis occur due to injuries to the esophagus caused by the accidental or intentional ingestion of chemicals, resulting in esophageal ulcers and esophageal stenosis. The extent of damage to the esophagus differs depending on the range and extent of chemical exposure and the type of chemical ingested, whether acidic or alkaline. Thus, the optimum treatment methods also vary. Treatment strategies for corrosive esophagitis include conservative treatment, such as balloon dilatation at the stenosis site, and surgical treatment. Cicatricial stenosis with corrosive esophagitis begins 2–6 weeks after chemical exposure. Stenosis develops gradually, and cicatricial stenosis gradually reaches completion after ~8–10 months [5]. Therefore, endoscopic dilatation for esophageal stenosis is often performed at an early stage after injury [6]. Though surgical intervention can also be performed at an early stage after injury, it is often performed in cases where endoscopic dilatation is unsuccessful or the stenotic lesion is extended [2]. Surgery involves esophageal resection, reconstruction or bypass. Since bypass surgery is reported to have a high rate of cancer development in the remnant esophageal mucosa [7, 8], esophageal resection is the most suitable treatment. Esophagectomy for corrosive esophagitis is performed using the transthoracic or transhiatal approaches [9, 10]. Since the first report of MIE by Cuschieri *et al*. [11] in 1992, the number of similar reports has gradually increased [3, 12, 13]. However, the position of the patient and ventilatory management methods vary across different facilities. Matsuda *et al*. [14] reported that they performed thoracoscopic esophagectomy for corrosive esophagitis with the patient in the prone position, but this can make it difficult to move the patient for thoracotomy in the event of an unexpected loss of a large volume of blood during surgery. Placing the patient in the semi-prone position, which is similar to the left lateral decubitus position, makes it possible to perform a rapid transition to the left lateral decubitus position and thoracotomy in cases of an emergency. Similarly, Cai *et al*. [15] reported perioperative management using differential pulmonary ventilation and bilateral pulmonary ventilation during thoracoscopic esophagectomy in a patient placed in the semi-prone position. Bilateral pulmonary ventilation has better PaO_2_ and PaCO_2_ intraoperative respiratory function than differential pulmonary ventilation, and it is also reported to have a shorter anesthesia induction time, reduced blood loss, shorter operation time with thoracoscopic procedures and shorter postoperative hospital stays.

In the present report, we describe a successful case of corrosive esophagitis treated with thoracoscopic esophagectomy with the patient in the semi-prone position using bilateral pulmonary ventilation. This technique could also be effective in the treatment of corrosive esophagitis.
